# Stability evaluation of compounded hydroxyurea 100 mg/mL oral liquids using a novel analytical method involving chemical derivatization

**DOI:** 10.1371/journal.pone.0270206

**Published:** 2022-06-24

**Authors:** Daphné Coache, Mihaela Friciu, Ruth Bernine Marcellin, Lola Bonnemain, Annie Viau, V. Gaëlle Roullin, Jean-Marc Forest, Grégoire Leclair

**Affiliations:** 1 Faculty of Pharmacy, Université de Montréal, Montréal, Québec, Canada; 2 Sainte-Justine University Hospital Center, Montréal, Québec, Canada; Laurentian University, CANADA

## Abstract

This study assessed the stability of six extemporaneously compounded hydroxyurea oral liquids stored at room temperature. Hydroxyurea oral liquids (100 mg/mL) were prepared using three different mixing methods (mortar, mixer or QuartetRx) from either bulk powder, capsule content, or whole capsules. Two brands of capsules were tested in this study. All formulations were stored at room temperature (25°C / 60% RH) in amber plastic bottles for 90 days and amber plastic syringes for 14 days. Physical stability was assessed visually, while chemical stability was evaluated using a stability-indicating high-performance liquid chromatography method. Chemical derivatization with xanthydrol allowed the retention of hydroxyurea on a reverse-phase column. At least 93.9% and 97.0% of the initial concentration of hydroxyurea remained after 90 days in bottles and 14 days in syringes, respectively. There were no visual changes in formulations over the study period. Changes in pH up to 1.6 units were observed after 90 days of storage and were explained most likely by an ammonium generating degradation pathway. Ammonium was quantified and remained within safe levels in each HU 100 mg/mL oral preparations. Hydroxyurea oral liquids were all stable for 90 days in amber plastic bottles and 14 days in amber plastic syringes.

## Introduction

Sickle cell disease (SCD) is an hemoglobinopathy characterized by red blood cell deformation and currently affecting about 5,000 Canadians including children [1]. This hereditary blood disorder causes chronic hemolytic anemia and vaso-occlusion leading to major complications, such as pain crisis and organ damages [2]. Hydroxyurea (HU), is a disease-modifying agent used to prevent SCD complications. HU, by altering the erythropoiesis cycle, increases the amount of fetal hemoglobin in red blood cells, thereby reducing hemolysis and sickling [3, 4]. Studies have shown several important clinical benefits for its use, especially in reducing acute painful episodes, blood transfusions, hospital stays, acute chest syndrome episodes and in extending life expectancy, both in adults and children [3–6].

In Canada, HU is only commercially available as capsules. It is well known that administration of a solid dosage form intended for adults is not an acceptable option in pediatrics as difficulty in swallowing could be an obstacle to treatment compliance [7]. Since HU is recommended for babies as young as 9 months of age, an increasing number of patients need HU liquid formulations [8]. In addition, liquid formulations offer greater dosing flexibility, which is essential for a treatment requiring regular dosing adjustment based on improvement of hemoglobin level, the weight of the patient and hematologic, renal or hepatic toxicity [6, 9].

Bioequivalence of HU liquids and capsules has previously been studied and was favorable to the administration of a compounded HU liquid in SCD-patients unable to swallow capsules [10]. The stability of 100-mg/mL HU oral liquids in different vehicles (Syrpalta, Ora-Blend and Ora-Blend SF) has already been reported in the literature [11, 12]. The preparation of the formulation in Syrpalta (registered trademark of Humco, TX, USA) is time-consuming and its stability is based on results obtained from a non stability-indicating method [11]. Oral liquids compounded in Ora-Blend or Ora-Blend SF (registered trademarks of Paddock Laboratories, MN, USA) were prepared from HU powder and stored in amber glass bottles for a period of 120 days [12]. However, in pharmacy practice, plastic is often preferred to glass because of its cost and weight. In the case of HU, a molecule classified in the National Institute for Occupational Safety and Health (NIOSH) list [13], safety should also be considered in the choice of material. Glass is more fragile and more at risk of breaking than plastic. Therefore, plastic containers should be preferred to limit the risk of unnecessary exposition to this NIOSH-listed antineoplastic agent. Similarly, capsules are far more accessible than bulk powder in pharmacies, which justifies evaluating their compounded stability in oral liquid preparations. A new technology has been proposed by the pharmaceutical industry to limit operators’ exposure to toxic molecules. QuartetRx (registered trademark of P&C Pharma, OH, USA) is an automated preparation system involving a single specialized bottle (S1 Fig) in which the compounded oral liquid is prepared and stored, without any transfer. QuartetRx is based on wet milling process and offers a cleaner and safer approach to compound oral liquids for NIOSH-listed molecules. This technology is designed to eliminate the aerosolization of powders during the compounding process.

The aim of this study was to assess the stability of 100-mg/mL HU oral liquids compounded from bulk powder, original and generic capsules in Ora-Blend. Different methods of preparation (mortar, mixer and QuartetRx) were tested to evaluate their impact on the stability. Each formulation was stored at room temperature in amber oral plastic syringes and amber plastic bottles for 14 and 90 days, respectively. Due to its chemical structure, the retention of hydroxyurea on a reverse-phase HPLC column is quite challenging. To obtain a stability-indicating HPLC-UV method, a chemical derivatization of hydroxyurea with xanthydrol was used. Xanthydrol is an agent, having a hydrophobic structure, that reacts with primary amides such as urea and hydroxyurea [14], thus improving their retention on nonpolar columns.

## Materials and methods

### Products

Methanol, isopropyl alcohol, acetonitrile (ACN), solution of hydrochloric acid (HCl) 1N, solution of sodium hydroxide (NaOH) 1N, solution of hydrogen peroxide (H_2_O_2_) 3% and HU standard powder were all purchased from Fisher Scientific (QC, Canada). Xanthydrol powder, monobasic potassium phosphate powder and ammonia assay kit (MAK310) were purchased from Sigma-Aldrich (QC, Canada). Original hydroxyurea capsules were purchased from Bristol Myers Squibb (lot #6463 exp: 06/2023, QC, Canada) and generic hydroxyurea capsules were purchased from Mylan Pharmaceuticals ULC (lot #90606A exp: 04/2022, ON, Canada). Ora-Blend (lot #0302344 exp: 06/2023) and HU powder (lot #00329–0219 exp: 02/2025) were purchased from Galenova (QC, Canada).

**Caution**: Hydroxyurea (HU) is hazardous (NIOSH—Group 1: antineoplastic agent) and should be handled carefully [13].

### Preparation of 100-mg/mL HU oral liquids

#### Mortar—Capsules or powder

Formulations produced with a mortar and pestle were prepared from either HU powder or capsule contents of both 500-mg original and generic capsules. Capsule contents (10 capsules) or HU powder (5.0 g) were reduced to fine powder in a mortar using a pestle. Then, Ora-Blend (50 mL) measured with a graduated cylinder was gradually added at controlled room temperature to the mortar using the geometric dilution principle while taking care of mixing after each addition.

#### Mixer—Powder

Formulations produced with a mixer were prepared only from HU powder. The appropriate amount of powder (5.0 g) was added to the graduated cylinder already containing a small portion of Ora-Blend. Then, enough Ora-Blend was added up to a final volume of 50 mL at controlled room temperature. The resulting mixture was transferred in a mixer (CB15, Waring Commercial, USA) and mixed at low speed for 5 minutes.

#### QuartetRx—Capsules

Formulations produced with the QuartetRx were prepared from whole capsules at controlled room temperature. Original or generic capsules (10 capsules) were placed in a QuartetRx patented bottle (S1 Fig) followed by the addition of Ora-Blend (50 mL), measured with a graduated cylinder. The mixing sequence was programmed as follows: run (12 min), pause (5 min), run (10 min), pause (5 min), run (10 min), pause (4 min).

### Forced degradation study

Specificity of the analytical method was evaluated by mixing equal amounts (0.5 mL) of a solution of HU powder in Ora-Blend (100 mg/mL) with each of the following solutions: water, 30% H_2_O_2_, 1N NaOH and 1N HCl. These samples were incubated at 70°C for 3 hours. A reference sample was prepared with water and kept refrigerated until analysis. After incubation, basified and acidified samples were respectively neutralized (0.5 mL) with 1N HCl and 1N NaOH. The same volume (0.5 mL) of water was added in the remaining samples. Extraction and derivatization procedures were performed as for stability samples. Overlap of degradation products with HU peak was evaluated visually on chromatograms.

### Stock solution preparations

A 0.02 M xanthydrol solution was prepared by transferring the appropriate amount of xanthydrol powder (39.6 mg) in a 10-mL volumetric flask. Isopropyl alcohol was added up to volume. The solution was then mixed for 5 minutes protected from light. The resulting solution was filtered (PVDF, 0.22 μm) and transferred in an amber glass container.

A phosphate buffer solution (pH 6.8) was prepared according to the method of preparation described in the United States Pharmacopeia—National Formulary (USP43-NF38, *Buffer Solutions* monograph) [15].

### Sample preparation: Extraction and derivatization

The extraction of HU was the first step involved in the sample preparation procedure. It was performed by mixing an aliquot of HU formulation (50 μL) with methanol (5.0 mL) for 20 s. This mixture was then centrifuged (2,135 g, Sorvall (RT6000D) registered trademark of DuPont, DE, USA) for 15 min. Derivatization was then directly performed in an amber HPLC glass vial. First, a portion of the clear supernatant (50 μL) obtained from the previous centrifugation was mixed with methanol (400 μL) and a freshly prepared solution of xanthydrol (0.02 M, 100 μL). Secondly, to allow the reaction to take place, HCl (1 N, 5 μL) was added to the vial. Once the vial content was mixed, it was left at room temperature for 10 minutes. Finally, pH 6.8 phosphate buffer (450 μL) was added to the vial to neutralize the pH, thereby stopping the derivatization.

### Chromatographic parameters

Chromatographic analysis was conducted using a Prominence UFLC HPLC (Shimadzu, QC, Canada) system consisting of a LC-20AD binary pump, a DGU-20A5 solvent degasser, an SPD-M20A multiple wavelength photodiode array detector, a SIL020AC HT refrigerated autosampler and a CTO-20AC column oven. Peak area and capacity factor were calculated with the aid of the HPLC LabSolutions 5.54 sp5 software (registered trademark of Shimadzu, QC, Canada). The chromatographic conditions of the validated analytical method are listed in Table 1.

**Table 1:** Chromatographic conditions for the quantification of hydroxyurea.

**Column**	ZORBAX* RX-C18 (5 μm, 4.6 x 150 mm)		
**Column temperature**	35°C		
**Mobile phase**	A: ACN		
	B: water		
**Flow rate**	1 mL/min		
**Gradient**	Time (min)	A (%)	B (%)
	0 à 8	28	72
	8.0 à 8.5	60	40
	8.5 à 13	60	40
	13 à 13.5	28	72
	13.5 à 20	28	72
**Detection wavelength**	240 nm		
**Run time**	20 min		
**Injection volume**	7 μL		

*Registered trademark of Agilent Technologies, DE, USA

### Calibration curve

To assess the linearity of the analytical method, a 5-point calibration curve covering a range from 80 to 120% of the targeted concentration (100 mg/mL) was prepared. A 120 mg/mL standard stock solution was prepared from HU powder in Ora-Blend and then diluted to achieve concentrations of 80, 90, 100 and 110 mg/mL. Extraction and derivatization were performed as for stability samples. The calibration curve was analyzed in triplicate on three different days to determine intra and inter-day precisions.

### Stability study design

Each HU compounded oral liquid was prepared in triplicate (n = 3). Formulations prepared using a mortar or a mixer were stored in amber plastic bottles (polyethylene terephthalate—PET, Richards Packaging, QC, Canada) with child-resistant caps, while the ones prepared using QuartetRx were stored in the patented amber QuartetRx plastic bottles (polypropylene, P&C Pharma, OH, USA). All bottles were stored at controlled room temperature (25 ± 2°C / 60 ± 5% RH–Thermo Scientific, Forma Environmental Chamber, OH, USA) for 90 days. Two 3.0-mL amber oral plastic syringes (NeoMed Inc, GA, USA) were filled with each formulation and stored in the same conditions for 14 days.

An aliquot (2.0 mL) was sampled on day 0, 7, 14, 30, 45, 60, 75 and 90 for samples stored in bottles, while sampling was stopped on day 14 for the ones stored in syringes. Bottles and syringes were shaken by hand prior to sampling.

On each time point, samples were evaluated for both chemical and physical stabilities. On the day of preparation and after 90 days of storage, pH measurements were performed to detect pH changes over time (pH 211, Hanna Instruments, QC, Canada).

### Data analysis

Results are presented as means ± standard deviations (SD) from triplicates. Initial concentration of oral liquids was reported to either the targeted concentration (100 mg/mL) or the quantified nominal concentration, depending on the method of preparation.

### Specifications—Physical stability

The physical stability of the formulations was visually assessed. The color and consistency of stability samples were evaluated at each time point.

### Specifications—Chemical stability

The chemical stability of HU formulations was evaluated using the stability-indicating HPLC-UV method previously described. Samples were considered stable if the concentration remaining from the initial concentration was not less than 90.0% on each timepoint [16, 17]. A change in pH of more than 1.0 pH unit was considered significantly different and triggered an investigation [18, 19].

## Results

### HPLC-UV method development

Initially, the HPLC-UV method described in the HU USP monograph (USP43-NF38) was tested for the quantification of HU [20]. When reproducing the USP method parameters, the retention time (t_R_) of HU was 4.9 minutes. The concern here was that the t_R_ of HU was the same as the time of elution of unretained compounds (t_0_). Capacity factor (k’) calculated according to the FDA guideline was 0 [21]. This indicated that HU was not retained on the stationary phase. Hence, the specificity of this analytical method could not be demonstrated for HU, a requirement for a stability-indicating HPLC-UV method.

Instead, to ensure optimal retention and separation of HU from impurities, excipients or degradation products, a method using xanthydrol derivatization was subsequently tested. This method had been previously used for the bioanalysis of hydroxyurea concentration in blood samples [22]. With this improved method, a capacity factor of 4.84 (t_0_ = 1.5 min; t_R_ = 7.26 min) was calculated, which showed that HU was well separated from unretained components.

### HPLC-UV method validation

The present analytical method was validated for accuracy, precision, linearity, and specificity.

The accuracy of the analytical method, described as the percentage of the real concentration recovered from the linear regression, was between 98.6% and 101.4% for all concentrations of the calibration curve. Coefficients of variation were not more than 0.1% and 0.5% for intra and inter-day precisions, respectively. The linearity of the method was determined by evaluating the R^2^ (0.9960) obtained from the calibration curve. The retention time of HU was 7.26 min.

**Fig 1:**
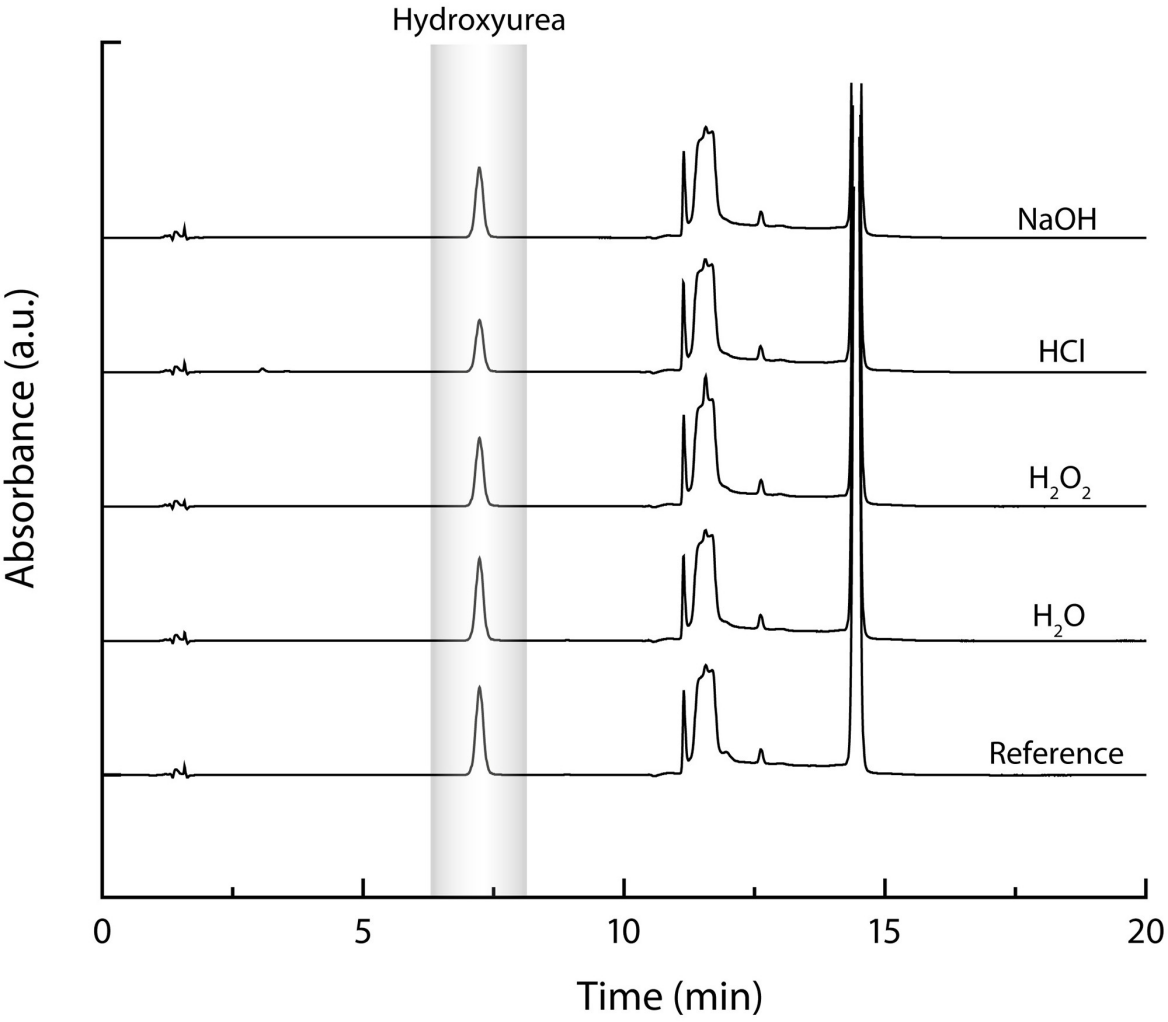
Representative chromatograms of forced degradation of HU.

In the presence of water, H_2_O_2_, HCl and NaOH, the proportion of remaining HU compared to the reference were of 93%, 78%, 59% and 80%, respectively. In all cases, no peak of degradation products interfered with the quantification of hydroxyurea (Fig 1).

### Chemical stability

**Fig 2:**
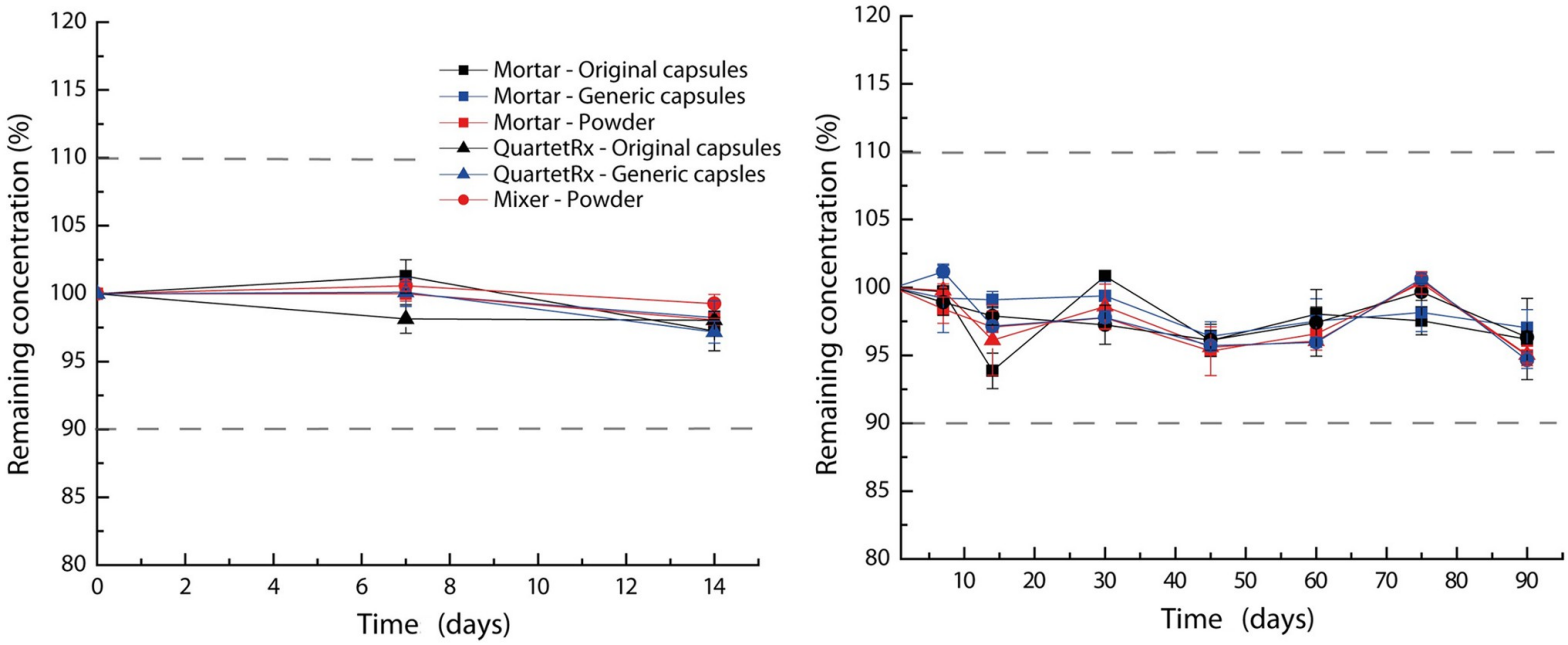
Chemical stability of HU oral liquids stored in amber oral plastic syringes (left) and amber plastic bottles (right). Initial concentrations (mg/mL): mortar—original capsules, 93.0 ± 0.5; mortar—generic capsules, 96.2 ± 1.4; mortar—powder, 97.2 ± 0.4; mixer—powder, 100.9 ± 0.7; QuartetRx—original capsules, 91.3 ± 0.7; QuartetRx—generic capsules, 93.6 ± 0.8. ●, mixer; ■, mortar; ▲, QuartetRx; red, powder; blue, generic capsules; black, original capsules.

All preparations had an initial concentration of not less than 90.0 mg/mL. The stability of hydroxyurea in all formulations, whether in plastic syringes or plastic bottles, during their respective study period, were not less than 90.0% of the initial concentration (Fig 2).

The pH of each formulation measured on day 90 was compared to their respective initial pH to note abnormal changes over time. Results are presented in Table 2. Noticeably, the pH of each formulation increased during the 90-day period, more markedly for formulations prepared from generic capsules and bulk powder, increasing of more than a 1.00 pH unit, regardless of the preparation method used. In all cases, pH remained slightly acidic.

**Table 2:** Comparison of pH values.

Preparation	pH on day 0 (mean ± SD)	pH on day 90 (mean ± SD)	Mean difference (pH units)
Mortar—Original capsules	5.39 ± 0.02	5.95 ± 0.01	0.56
Mortar—Generic capsules	4.18 ± 0.01	5.61 ± 0.00	1.53
Mortar—Powder	4.21 ± 0.05	5.62 ± 0.02	1.41
Mixer—Powder	4.26 ± 0.02	5.88 ± 0.14	1.62
QuartetRx—Original capsules	5.45 ± 0.00	5.92 ± 0.01	0.47
QuartetRx—Generic capsules	4.48 ± 0.01	5.55 ± 0.01	1.07

After a storage period of 90 days at room temperature, changes in excess of 1 pH unit were observed (Table 2). The pH of formulations prepared from original capsules, regardless of the method of preparation, was initially higher and less variable over time than the ones prepared from generic capsules or bulk powder. Formulations prepared from generic capsules or powder were subject to increased pH values (more than 1 pH unit). All preparations remained slightly acidic even after 90 days (pH 5.55 to 5.95) and therefore did not substantially change the ionisation state of hydroxyurea (pKa = 10.6). Possible causes of these pH changes were therefore investigated.

**Fig 3:**
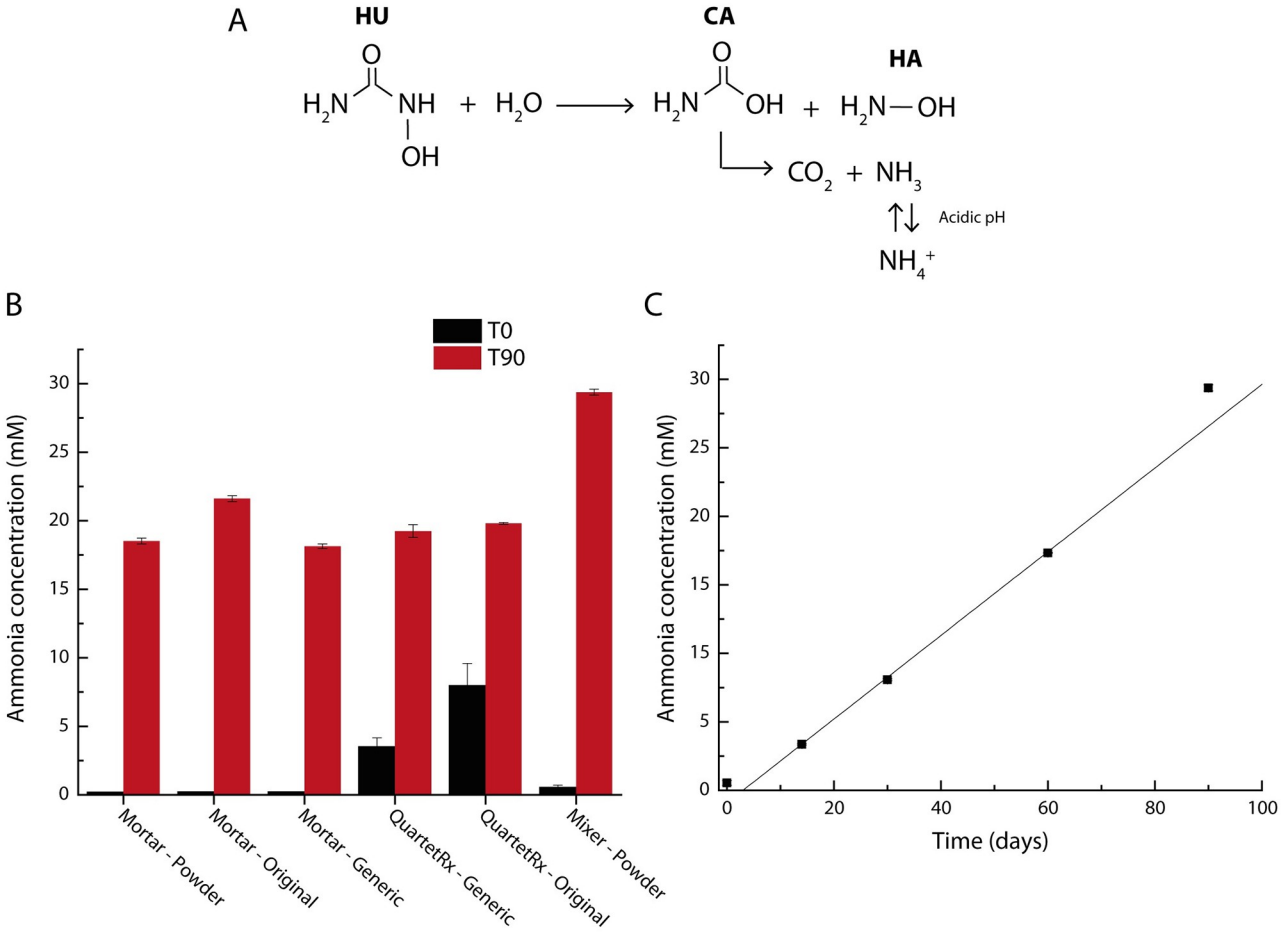
Formation of ammonium in HU oral liquids. Reaction of hydrolysis of hydroxyurea in aqueous liquids (A). Comparison of ammonium concentration in oral liquids at T0 and T90 of the stability study (B) and kinetic of ammonium formation in an HU oral liquid (C). HU, hydroxyurea; CA, carbamic acid; HA, hydroxylamine. The error bars correspond to the minimum and maximum measurements.

The most plausible cause for the pH changes observed in this study involves HU hydrolysis resulting in the formation of carbamic acid and hydroxylamine products (Fig 3A). Carbamic acid is then rapidly converted to carbon dioxide and ammonia [23, 24]. Ammonia (NH_3_, pKa 9.25) is then completely converted to ammonium ion (NH_4_^+^) at pH lower than 6 resulting in the observed pH increase. Even if no significant pH changes were observed for oral liquids prepared from original capsules, this reaction might also have occurred during the stability study. In fact, original capsules contain dibasic sodium phosphate and citric acid (S1 Table) [11], which could likely have acted as a buffer system on preparations made from these capsules.

**Fig 4:**
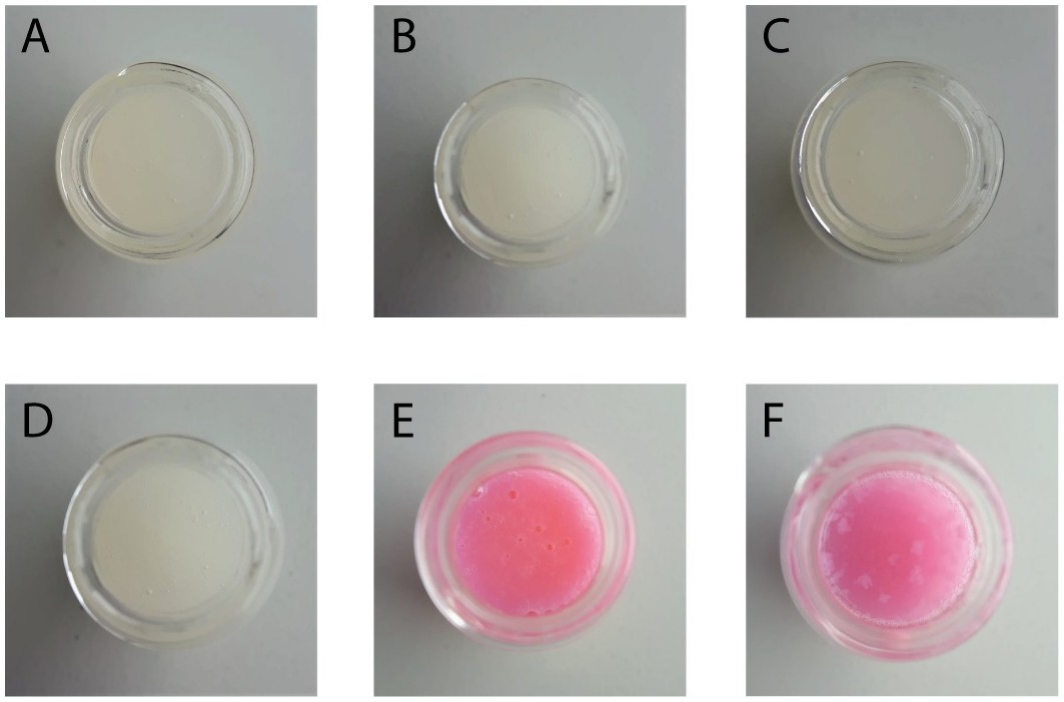
Appearance of HU oral liquids. A, Mortar—Original capsules; B, Mortar—Generic Capsules; C, Mortar—Powder; D, Mixer—Powder; E, QuartetRx—Original capsules; F, QuartetRx—Generic capsules.

Given the high HU concentration in oral liquids (100 mg/mL), our hypothesis was that the observed degradation of 3% to 6% HU after 90 days of storage (Fig 4) could account for observed pH changes.

To confirm this hypothesis, the quantification of ammonium in the oral liquids was performed. A commercial kit from Sigma-Aldrich using *o*-phthalaldehyde and fluorescent quantification (λ_ex_ = 360 nm, λ_em_ = 450 nm), was used. First, a sample of each formulation was evaluated (in duplicate) at T0 and T90 timepoints, to compare ammonium levels at the beginning and the end of the study (Fig 3B). To evaluate the kinetics of ammonium production over time, the formulation prepared from powder with a mixer was evaluated at T0, T14, T30 and T60 and T90 timepoints (Fig 3C).

For all samples, on day 0, ammonium concentration was less than on day 90. Samples prepared with the QuartetRx had an initial ammonium concentration higher than samples prepared with either a mortar or a mixer. This difference could be explained by a release of energy during QuartetRx preparation process, resulting in the generation of ammonium from hydroxyurea hydrolysis on day 0. After 90 days of storage, ammonium concentrations in HU oral liquids were all measured between 18.14 and 29.38 mM (Fig 3B). Ammonium production kinetics was evaluated for an HU oral liquid and was assumed to be similar for other HU sources and preparation methods. It is possible to observe that ammonium formation was linear over time (Fig 3C). From the stoichiometry of the reaction proposed in Fig 3A, we can estimate the amount of HU degraded to generate such ammonium concentration. From the highest ammonium measurement (29.38 mM), this would correspond to 2.2% of the initial HU concentration. This agrees with HPLC measurements provided during chemical stability study, remaining below 10.0% of loss from the initial concentration.

### Physical stability

All preparations from capsule contents or bulk powder were translucent and white, while the ones prepared from whole capsules were translucent and pink. The formulations produced with the mortar or the mixer were all identical in aspect (Fig 4A–4D). A difference in the appearance of liquids compounded using QuartetRx was observed (Fig 4E and 4F). Undissolved pieces of capsules remained in suspension for formulations prepared from the generic capsules. However, both oral liquids, from original and generic capsules, produced with the QuartetRx were treated the same. The presence of undissolved pieces of capsules could be resolved with an additional cycle of QuartetRx. The visual appearance of all formulations, independently from the method of preparation or the source of HU, did not change after 90 days of storage.

## Discussion

### Physical stability: QuartetRx

A difference in the appearance of formulations prepared from original and generic capsules using the QuartetRx has been noted. This problem seemed to be related to the capsule shell dissolution. At the end of the programmed QuartetRx cycle, undissolved pieces of generic capsule shells were still in suspension. The composition of both original and generic capsules is quite similar (S1 Table) and, to our knowledge, could not explain *a priori* the differences observed since both are gelatin capsules [25, 26]. However, method of fabrication and storage of hard gelatin capsules [27], pH of the oral liquid, excipient in capsule contents, are all possible causes of the discrepancy in dissolution rates observed between original and generic capsules. Unfortunately, formulations prepared from generic capsules looked less attractive than the one prepared from original capsules, and the presence of small pieces of capsule shell could limit its acceptance in pediatrics.

This observation highlights the importance of choice regarding ingredients, vehicle, method of preparation, when compounding an oral formulation. It should not be assumed that two capsules presenting similar composition will give a final product displaying a same appearance. Pharmaceutical compounding is complex, even more when the whole capsule is used. It is the first time that a capsule shell is incorporated into a liquid formulation and this difference in dissolution could be experienced with other active ingredients from capsules if QuartetRx was to be used as described in this publication.

### Concentration accuracy of HU oral liquids

Initial concentrations of HU oral liquids, except for the ones produced with a mixer, were noticeably lower than the nominal concentration of 100 mg/mL, ranging from 91% to 96% of the target concentration. At first, the volumes occupied by the HU, excipients and capsule shells were deliberately not considered for the formulations produced with the QuartetRx or using a mortar. Indeed, these preparations were simply made by adding a volume of 50 mL of Ora-Blend rather than adding the required volume of Ora-Blend to achieve a final preparation volume of 50 mL. This choice was made to limit the operator’s exposure to the NIOSH-listed antineoplastic agent by limiting the manipulation of hazardous products. Based on the density calculations (Eq 1), it was possible to calculate, a posteriori, the exact volumes of Ora-Blend required to complete the preparations to a final volume of 50 mL and achieve a 100 mg/mL HU concentration:

$$V_{OB}\;\left(100\;mg/mL\right)=50\;mL-\left(\frac{M_{OB}\left(50\;mL\right)+M_{solids}}{\rho_{\text{f}ormulation}}\right)-50\;mL)$$ (1)

where V_OB_ is the volume of Ora-Blend required to achieve a concentration of 100 mg/mL, M_OB_ is the mass of 50 mL of Ora-Blend (56.94 g), M_solids_ is the mass of solids added in oral formulations either from powder, capsule content, or entire capsules, and ρ_form_ is the density of HU oral formulations compounded. V_OB_ was calculated for all HU formulations (Table 3), except the one prepared from powder using a mixer, for which the final volume was directly adjusted to 50 mL.

**Table 3:** Exact volume of Ora-Blend required to complete each preparation (50 mL) to achieve an HU final concentration of 100 mg/mL.

Preparation	ρ_form_^a^ (g/mL)	M_solids_^a^ (g)	V_OB_ (mL)
Mortar—Original capsules	1.1755	6.022	46.4
Mortar—Generic capsules	1.1727	5.057	47.1
Mortar—Powder	1.1700	5.058	47.0
QuartetRx—Original capsules	1.1694	6.946	45.4
QuartetRx—Generic capsules	1.1620	5.787	46.0

^a^ n = 3

From these results, it was possible to calculate the theoretical HU concentration of the formulations listed in Table 3. The accuracy of the calculated concentration was between 97.08 and 100.36% of their respective concentration measured by HPLC-UV on day 0 of the stability study.

### Ammonium and hydroxylamine degradation products

Ammonium is produced endogenously by several organs, such as in the gut during urea and protein breakdowns by bacterial ureases and proteases, respectively. This production is estimated to 40–60 mg/kg per day for a 70-kg adult [28]. Humans are also exposed to exogenous sources of ammonium, mainly from the consumption of water and food. For example, daily exposure to exogenous ammonium is estimated to be 18 mg for adults [29]. Unfortunately, this value is not available for children, but ammonium would be metabolized as adults. In healthy humans, ammonium is largely absorbed in the gastrointestinal tract, metabolized to urea in the liver, and finally, excreted in the urine [29, 30].

The study of oral exposure to ammonium in children, as in adults, is very limited [31]. The only interpretation of ammonium exposure in children is from water, where a risk assessment was performed for an ammonium level of 0.5–5 mg/L [28], which was shown to be of no safety concern. The main reason was that the absorption of ammonium from water was not significant compared to the amount of ammonium endogenously generated in the gastrointestinal tract. It is estimated that a 2-year-old child (12 kg, or 26 lbs) consumes about 1.3 L of water per day. The exposure to ammonium from water would therefore be of 0.65–6.50 mg per day. This basal value can be compared to the ammonium exposition related to HU oral liquids for a child having the same characteristics of age and weight. HU is administered in pediatrics at a dose of 20 to 30 mg/kg per day. Considering the worst-case scenario, *i*.*e*. of that a 30 mg/kg dose of a 100-mg/mL HU oral liquid prepared from powder using a mixer and stored at room temperature for 90 days, this child would ingest 1.90 mg of ammonium. The exposure would not be greater than that from water, for which no safety concerns have been raised [28].

**Fig 5:**
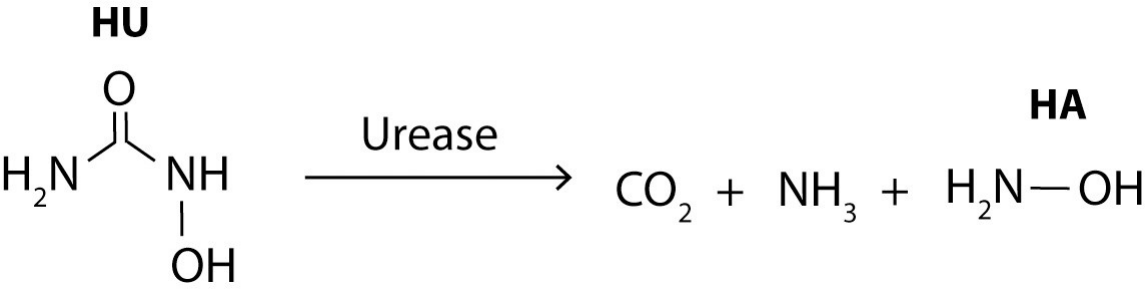
Urease mediated HU metabolism pathway.

Although HU has been used for several decades, its metabolism remains unclear. The major degradation pathway occurs in the liver (between 50–60%), with a minor degradation pathway involving bacterial urease in the gastrointestinal tract [25]. The extent of this metabolic pathway is not fully understood but there is no doubt that it also generates ammonium once ingested (Fig 5). Therefore, ammonium generated from the metabolism of all types of ingested HU formulations would also contribute, and probably to a much greater extent.

The presence of ammonium in HU oral liquids also suggests hydroxylamine (HA) formation. From the equimolar reaction shown in Fig 3A, assuming that all or most of the carbamic acid (CA) is converted to ammonium, at most 29.38 mM of HA would be produced in oral liquids. HA is a product of human cell metabolism [32]. However, its potential toxicities have raised some concerns among the scientific community. The World Health Organization (WHO) have calculated a *permissible daily exposure* (PDE) for HA based on preclinical carcinogenicity studies. The PDE for HA is 2 μg per day [33]. During the metabolism of HU by urease (Fig 5), HA is also produced [23]. We don’t know yet the extent of the urease activity on HU metabolism, since HU is mainly (about 50%) metabolized in the liver and the kidney by different mechanisms [34]. However, a clinical study has demonstrated that HA is formed after HU administration by the intermediate of urease [35]. They have estimated that 1 to 10% of the administered HU dose was converted to HA. The amount of HA produced from HU metabolism is therefore largely above the WHO lifetime PDE. In the worst-case previously mentioned for ammonia, a child (12 kg) would ingest 3.49 mg of HA from an HU oral liquid stored for 90 days at room temperature. For the same patient, 3.6 to 36 mg of HA, corresponding to 1 to 10% of the administered dose, would be generated during HU metabolism. From these results, no additional safety concerns are raised from this degradation product, since the patient would inevitably be exposed to HA, in similar amount, during metabolism.

Because the potency of HU oral liquids did not decrease below 90.0% of their initial value, it is unlikely that their overall efficacy would be affected. Although the formation of potentially toxic degradation products could have raised safety concerns, we demonstrated that patients’ exposition to ammonium and HA is inevitable with HU treatment. Degradation of HU in compounded preparations can be tolerated and, to limit the risks, the latter should be used before the beyond-used date.

It is important to note that HU is a drug used in life-threatening diseases, such as cancers and SCD. When it is not dosed properly, HU is considered has a toxic agent [13]. However, the benefits encountered by this treatment are greater than its toxic effects at therapeutic doses [36]. There are also several studies that demonstrate long-term safety of HU [37, 38]. The treatment is usually well tolerated without any serious complications or secondary malignancy.

This situation demonstrates the importance of investigating potential degradation mechanisms through different methods, and not only to base the beyond-use date on the quantification of the sole active ingredient by HPLC-UV. Aqueous formulations, such as oral liquid formulations, are more likely to show instabilities. Systematically studying all degradation pathways of active ingredients which could lead to toxicity would be an interesting option to ensure the safety of compounded preparations.

### Global stability of oral suspensions

All preparations remained stable for 90 days at room temperature in plastic bottles and 14 days in plastic syringes according to USP requirements.

This is the first study reporting the use of QuartetRx to extemporaneously compound oral liquids with whole capsules. This method of preparation could be used for the preparation of other antineoplastic drugs in oral liquid forms. However, concentration inaccuracies related to the method of preparation, for safety purposes, and differences in formulation appearance between capsule brands should be individually addressed for further development.

## Conclusion

Stability of HU 100 mg/mL oral formulations in the readily available Ora-Blend vehicle and stored in amber plastic bottles and syringes was assessed with a new HPLC-UV stability-indicating method based on derivatization of HU with xanthydrol. Original capsules, generic capsules and bulk powder were the three different sources of HU. Different methods of preparation were also investigated and discussed, including a safer approach for NIOSH-listed products. Although a probable ammonium and HA production during storage was detected and reported for the first time, their levels were deemed safe for children use. Consequently, a beyond-use date of 90 days in amber plastic bottles and 14 days in amber plastic syringes was assigned to all studied HU 100 mg/mL formulations.

## Supporting information

S1 Fig QuartetRx specialized plastic bottle. (TIF)

S1 Table List of ingredients for hydroxyurea original and generic capsules. (PDF)

S1 Appendix HPLC results. Excel file containing all reported HPLC results.
(XLSX)

S2 Appendix HPLC results analysis. Excel file containing all analyses performed from HPLC results.
(XLSX)

S3 Appendix Measurements and results analysis. Excel file containing all results related to the pH, concentration determination (density), and ammonia quantification.
(XLSX)
