# Supplementary figures and images for: Stability evaluation of compounded hydroxyurea 100 mg/mL oral liquids using a novel analytical method involving chemical derivatization
Source: PLoS One. 2022 Jun 24;17(6):e0270206. doi: 10.1371/journal.pone.0270206 (PMC9231814; doi:10.1371/journal.pone.0270206)

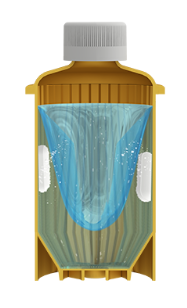

Supplement: S1 Fig — (TIF) [file pone.0270206.s001.tif]
