# Supplementary material for: Stability evaluation of compounded hydroxyurea 100 mg/mL oral liquids using a novel analytical method involving chemical derivatization
Source: PLoS One. 2022 Jun 24;17(6):e0270206. doi: 10.1371/journal.pone.0270206 (PMC9231814; doi:10.1371/journal.pone.0270206)
Supplement: S1 Table — (PDF) [file pone.0270206.s002.pdf]

**Original capsules**	**Generic capsules**
Hydroxyurea	Hydroxyurea
Lactose monohydrate	Colloidal silicon dioxide
Magnesium stearate	Magnesium stearate
Citric acid anhydrous	Gelatin[a]
Sodium phosphate dibasic anhydrous	Titanium dioxide[a]
Erythrosine[a]	D & C Red #28[a]
Gelatin[a]	FD & C Blue #1[a]
Titanium dioxide[a]	FD & C Red #40[a]
Indigo carmine[a]	D & C Yellow # 10[a]
Yellow iron oxide[a]	Black SW-9008/SW-9009[a]

[a] Capsules shell
